# “Cold Hard Cash”: The Value of Direct Unconditional Compensation for People With Lived Expertise of Homelessness

**DOI:** 10.1111/hex.70708

**Published:** 2026-05-29

**Authors:** Jeff Rose, Steffine Amodt, Yixiao Burke, Sarah L. Canham, Natalie Clark, Shawn Clay, Herbert Elliott, Rob Ferris, Ruby Gary, Lawrence Horman, Danielle Littman, Maygan Martinez

**Affiliations:** ^1^ College of Health University of Utah Salt Lake City Utah USA; ^2^ Salt Lake Valley Lived Expert Task Force Salt Lake City Utah USA; ^3^ College of Social Work University of Utah Salt Lake City Utah USA; ^4^ College of Humanities University of Utah Salt Lake City Utah USA

**Keywords:** community‐engagement, compensation, equity, gift cards, homelessness, lived expertise

## Abstract

**Introduction:**

Academic research projects that are oriented toward social and environmental justice have long recognized that participant compensation is part of reciprocal, just, community‐engaged relationships. Participant compensation acknowledges and accounts for people's expertise, skills, time and effort associated with contributing to research outcomes. However, a combination of institutional barriers and researcher hesitancy often limits the types of compensation used by research teams, and thus received by research participants. In keeping with best practices for research that engages with marginalized communities, this study considers the importance of direct unconditional cash compensation for people with lived expertise of homelessness.

**Methods:**

This research presents the results of semi‐structured interviews, group dialogues, and public presentations from people with lived experience of homelessness.

**Results:**

Our findings demonstrate (a) the epistemological and practical benefits that research participants receive from direct cash transfers, and (b) the institutional and moralistic barriers associated with direct cash transfers.

**Discussion:**

We conclude with a series of practical recommendations for researchers looking to incorporate this prioritization of justice‐focused compensation into their research projects.

**Patient or Public Contribution:**

People with lived experience of homelessness were collaborators, researchers and participants in this research, and were thoroughly involved in all parts of the research process, from ideation and study design to analysis and manuscript preparation.

## 1 Introduction

Compensating individuals for participating in research is a long‐standing practice in many academic and community‐based studies [1]. While compensation is often framed as a matter of fairness and basic equity, it is also inextricably linked to recruitment, risk and the logistical challenges of engaging participants, particularly those from marginalized or impoverished populations or those participating in studies perceived by the participants as particularly difficult or challenging [2]. Compensation is the primary reason given to justify payment for research participants, but is guided by incentivization, recruiting difficulties, inconvenience, risk and other factors [3]. This layered rationale highlights the need for a more participant‐centred and ethically grounded understanding of compensation practices.

Conversations about research compensation are inseparable from the broader politics of knowledge production. In a classic text on critical methods, Tuhiwai Smith [4] critiques institutional research norms rooted in colonial power dynamics. She emphasizes that decisions about who is paid, how much, and in what form are not politically or morally neutral but are embedded in histories of control and epistemic and material violence [4]. From this perspective, offering payment may be more than a practical choice; it can signal recognition of autonomy and an attempt to address structural inequalities embedded in research. However, such payments are not a cure‐all, as the structures, discourses, assumptions and implications of researcher–participant relationships still require further examination.

Equity‐focused shifts in community‐engaged and participatory methods have raised questions of justice [5], particularly in research involving people with lived experiences of homelessness [6]. Lived experience includes both those who have historically experienced homelessness and those currently living it. These populations face heightened precarity and vulnerability, yet are often uncompensated or undercompensated in academic or medical research [7]. Participants are sometimes offered restrictive compensation, often gift cards, which present multiple problems. Functionally, gift cards may not meet immediate needs (e.g., rent, fuel, transportation) or circumstances (e.g., store requiring transportation, online retailer needing internet and address). Ethically, gift cards may limit participants’ agency in deciding how to use funds [8]. Given these concerns, this study examines participant compensation by exploring perceptions of direct cash payments alongside procedural and ethical issues raised by supporting institutions.

The purpose of this study is to understand how direct cash payments for research participants are perceived by those participants, and to characterize the procedural practices and concerns from academic institutions administering these payment processes. Our research is driven by the following questions: *How do individuals with lived experience of homelessness perceive and experience current forms of compensation in community‐engaged research, and are there are more effective and ethically grounded practices? What are the ethical, practical and structural implications and complications of institutional compensation practices?* We examine these dynamics particularly in the context of community‐based research with individuals with lived experience of homelessness [9], a population that continues to face social and structural exclusion in a variety of contexts [10], yet whose insights are vital to meaningful and justice‐forward policies and service interventions aimed at supporting unhoused persons. Our research is designed to provide an analysis of compensation practices that are specifically participant‐centred, the ethical implications for future research practices and the methodological and institutional insights for equity‐driven reform.

## 2 Literature Review

### 2.1 Compensation Ethics and Institutional Practices

The ethics of participant compensation have been debated in human‐subjects research, especially as studies expand into community‐based settings. Compensating community experts in research partnerships is widely regarded as preferred practice [11], yet payments are also viewed as potentially coercive for recipients [12]. Traditionally, compensation has been justified as offsetting the time, inconvenience and discomfort of participation. However, this rationale is shaped by institutional priorities, disciplinary norms, and moral assumptions about participant vulnerabilities and capacities [2]. Some argue there is a moral obligation to pay participants based on social beneficence alone [13], ensuring participation yields immediate benefit. This highlights how notions of ethical fairness are entangled with recruitment efficacy and productivity [14, 15].

A series of Canadian case studies supports paying insecurely housed participants with cash. Direct or unconditional cash transfers are a trust‐based homelessness intervention that prioritizes participant autonomy over typical eligibility constraints [16]. Evaluations of these pilots show that direct cash compensation increases the likelihood of improved housing stability, financial independence, and well‐being. These case studies also highlight the potential of cash transfers to reshape service delivery by offering flexible financial support instead of shelter‐based interventions [17, 18]. Advocacy videos and webinars further stress that programme success requires broader buy‐in and investment from funders, policymakers and service providers [16]. In effect, what is required is multifaceted support for cash payments from all parties to encourage, facilitate and ultimately ensure that ethical compensation is a part of the research and community engagement process with people with lived expertise of homelessness. Together, these resources show growing theoretical and practical support for direct cash transfers as a scalable, dignity‐preserving approach that reduces financial vulnerability and fosters housing stability while guiding rigorous implementation and evaluation.

Academic institutions often impose restrictions on how participants may be compensated. Equitable practices that address participant needs vary across projects and generally require stronger institutional guidance [2]. Direct cash payments are commonly discouraged or banned in favour of gift cards, perceived as easier to track and control [19]. Yet such preferences can create unintended consequences: gift cards may limit autonomy, be unusable without identification, and expose recipients to stigma, especially when tied to stores that fail to meet basic needs or where discrimination has occurred [1]. Although framed as protective for institutions and recipients, these practices can act paternalistically, implying distrust in participants’ ability to make appropriate choices [20, 21].

This tension becomes especially acute in research with marginalized groups such as people experiencing homelessness [6]. Concerns about the misuse of cash often reflect societal bias rather than empirical evidence; ‘misuse’ can serve as a euphemism for assumptions about what people ‘need’ instead of respecting recipients’ self‐determination. Contrary to common beliefs, cash given to people experiencing homelessness is typically used for housing, food, transportation, bills, vehicle maintenance and health needs, despite moralistic fears that funds will go toward drugs, alcohol, prostitution or other socially disfavoured activities [22, 23]. Additionally, for many who lack banking access, identification, stable addresses or internet access, gift cards can be impractical or useless [24]. People with lived experience of homelessness may face specific barriers to using gift cards, including transportation limits, lack of online ordering or delivery options or cards that cannot meet basic urgent needs. While most individuals experiencing homelessness identify immediate needs such as housing, employment, transportation, food, health services and social support [25], gift cards often offer limited usefulness. Thus, compensation mechanisms must be assessed not only for institutional convenience but for their fit with participants’ real‐life contexts and needs [2, 23]. Critical perspectives on these issues suggest that much of the engagement with people living through homelessness is unlikely to be successful if it is not supported by a level of basic human care and consideration that may be lacking in homelessness services sectors [26].

### 2.2 Community‐Based Research and Participatory Ethics

Community‐based participatory research (CBPR) frameworks provide a useful model for rethinking compensation within a broader ethic of engagement [11]. CBPR seeks to reduce power imbalances by co‐producing knowledge and ensuring shared research benefits [27, 28]. Such community‐engaged planning has long existed; for people experiencing homelessness, co‐developed research guidelines emphasize agency, reciprocity and empowerment [9]. Community engagement with lived experts has been critiqued when it has been employed in haphazard or seemingly insincere ways [29], pointing to the necessity of guidelines that are core to responsibly and ethically engaging with the phenomenon at hand. These guidelines call for people with lived experience to serve on paid planning boards, consultation teams and assessment evaluations [30, 31]. Here, compensation functions not only as an incentive but as recognition and redistribution, valuing experiential expertise and addressing historical and ongoing extractive research practices.

Further, research with insecurely housed populations must contend with a variety of situationally contingent issues, including those of mobility, trauma and institutional distrust, necessitating an intersectional approach that recognizes and engages seriously with issues like racism, misogyny, homophobia and colonialist displacement [10, 32]. Many individuals have experienced coercion or surveillance in previous encounters with service systems, which can make these individuals wary of participating in any research at all, and even more so if the benefits are unclear or the compensation inadequate [33]. Offering direct cash payments may alternatively serve as a mechanism to build researcher‐participant trust and, in some cases, may be seen as more respectful than conditional or restrictive forms of compensation [22, 23].

A participant‐centred approach to compensation also aligns with evolving social attitudes. Popular perceptions toward homelessness are changing [34], with growing awareness of the structural causes of homelessness and a shift toward empathetic, human‐centred policy solutions. While stigma and discrimination still shape the experiences of many housing‐insecure individuals [10], research indicates that there is increasing public and institutional commitment to making homelessness rare, brief and non‐recurring [35, 36], for the benefit of the entire community, regardless of one's housing status [37]. Meaningful engagement with people experiencing homelessness in the research process, including fair and flexible compensation, is essential to achieving this vision [38].

### 2.3 Epistemic Justice and Lived Experience

Our research team includes nine Lived Expert Task Group members with diverse experiences of homelessness and four university‐based researchers committed to understanding and addressing homelessness from multiple perspectives. We (the authors) share a commitment to honouring and elevating the voices of those most closely connected to the issue [25]. The (dis)connection between lived experience and lived expertise in community‐based research carries practical and epistemological implications for justice [39, 40]. This disconnection is evident when those calling for systemic change (e.g., academic, securely housed researchers) are not the individuals most affected by such changes (e.g., insecurely housed people, those with lived experience of homelessness). Including people with firsthand experience not only strengthens research findings but also challenges academic hierarchies about who counts as a ‘knower’ [4, 41].

Compensation practices can either reinforce or challenge these hierarchies. When people with lived experience of homelessness are consulted but unpaid—or paid in ways that do not meet their needs—it implies their insights are only partly valued. In contrast, equitable compensation affirms that their time, expertise and contributions are essential [6, 31] and signals a readiness to revise institutional procedures that may be outdated, overly cautious or misaligned with participatory justice [2]. Advisory groups of people with lived expertise may compensate members for participation, preparation, transportation and related costs, and often provide food at meetings [6, 10]. Forms of compensation, including gift cards, cheques, automatic transfers or cash, may vary by group or individual preference [6].

In sum, the literature suggests that direct cash payments for participants, especially those experiencing homelessness, are both ethically and practically preferable in many cases. Direct cash payments align with contemporary movements toward participatory ethics, address logistical and practical barriers and serve as a modest form of reparative justice within the research process. Yet, institutional resistance remains a significant hurdle. Understanding both the perspectives of participants and the procedural concerns of academic institutions is therefore crucial for developing more responsive, respectful and effective research compensation policies.

## 3 Methods

This study employed a qualitative research design grounded in interpretivist and critical participatory paradigms to explore individual and institutional perspectives related to understanding the implications of direct cash transfers to research participants with lived expertise of homelessness. Data for this study were collected from three primary sources: (a) in‐person meetings with a lived experience group, (b) public presentations delivered by the same group and (c) in‐person semi‐structured interviews with two academic account administrators (an operations manager and a lead grants specialist). These methods were chosen to enable a nuanced understanding of lived experiences, public narratives and organizational contexts [5].

This study was reviewed and approved by the Institutional Review Board (IRB) at the University of Utah, ensuring that all procedures complied with ethical standards for research involving human participants. All participants provided informed consent prior to participation. For the lived experience group, informed consent was obtained at the outset of the research period and reiterated prior to any audio or video recording. For public presentations, consent was obtained from presenters to use recordings for research purposes. The academic institutional interviewees were also informed of the study's purpose and provided verbal consent before participating in the interview. Participants were assured of their right to withdraw at any time, and confidentiality was maintained through anonymization of transcripts and secure data storage.

### 3.1 Data Collection

Thirteen two‐hour in‐person lived experience group meetings were held in a community‐based setting with individuals who had direct, personal experience with homelessness. The parent study aimed to develop guidelines for best engaging lived experts in research; our sub‐aim concerning cash payment emerged as a key focus after lived experts strongly urged university‐based researchers to elevate this issue. During group meetings, participants were compensated $60 USD for the two‐hour meetings (e.g., at a rate of $30 USD per hour). The compensation rate was determined to reflect fair remuneration for participants’ time and expertise, align with local living wage standards, and acknowledge the value of lived experience as a form of specialized knowledge within the research process. While we never systematically inquired about participants’ comfort with that compensation rate, we anecdotally heard that the amount felt appropriate for their time and engagement with these complex and personal issues.

Group members reflected diverse ages, genders, races and experiences of currently or previously living insecurely housed. University‐based researchers participated in multiple roles throughout the project and in group meetings, ranging from collaborative partners to nonintrusive observers. Meetings were audio‐recorded and documented with detailed field notes capturing verbatim quotes and key points. These interactions offered in‐depth insight into group dialogue, collective meaning‐making and interpersonal dynamics. Ethical considerations, including confidentiality and voluntary participation, were prioritized throughout.

To complement data from the lived experience group discussions, we included data from recorded public presentations conducted by the same group in collaboration with the academic team. These events, designed to communicate to service providers, community members, university researchers and policymakers, provided insight into how participants experienced direct cash payments in public and advocacy contexts. Audio recordings and transcripts were analyzed to assess differences in tone, content and purpose compared with the in‐person group discussions [42].

Finally, two semi‐structured interviews were conducted with academic account administrators—an operations manager and a lead grants specialist—to gather contextual information on direct cash transfers from an organizational perspective. Using a modified Seidman [43] approach, the interviews explored administrative procedures, systemic challenges and institutional responses relevant to the lived experience group's engagement and compensation. This approach involves three sequential categories allowing respondents to communicate their (1) focused history with the issue, (2) perceptions, insights and experiences and (3) reflections and meanings regarding the issue. The same open‐ended questions guided both interviews for consistency, while the semi‐structured format allowed flexibility to pursue clarifying or exploratory topics [44, 45]. Interviews were audio‐recorded, transcribed and analyzed alongside other data sources.

### 3.2 Data Analysis

All collected data were transcribed and analyzed using thematic analysis. Codes were refined iteratively through a constant comparison method, and themes were developed through collaborative review [46]. Connection, overlap and overall triangulation across the three data sources enhanced the credibility of the findings [47], while analytic memos and researcher reflexivity supported rigour and transparency throughout the analysis [48]. Additionally, having multiple roles in the research process, from researcher to participant to author, increases the analytical depth of this research [49].

## 4 Findings

Findings were organized into three categories, each with its own sub‐categories: (1) benefits of compensation; (2) benefits of direct cash transfers; and (3) barriers to implementing cash payments. Table 1 lists these themes as well as subthemes and a representative quote for each; additional direct quotes and further analyses are provided in the subsequent sections.

**Table 1:** Themes, subthemes and representative quotes.

Theme	Subtheme	Representative quote
Benefits of compensation	Respect and equity	‘[Compensation is] not just a transaction. It's a statement of respect, recognition, and equity’. (Lived Expert)
	Honours lived experience as expertise	‘There is nobody at this table that doesn't have a minimum equivalent of a master's [degree] in the School of Hard Knocks’. (Lived Expert)
Benefits of direct cash transfers	Practicality of cash honoraria	‘A lot of our people…may not have a bank account, may not have ID, may not have transportation to get to a bank…’ (Lived Expert)
	Participant autonomy and empowerment	‘Nobody tells you what to do with your paycheck, so why is that different for me’? (Lived Expert)
Barriers to implementing cash payments	Institutional ‘red tape’	‘We used these folks’ words and experiences to make the case… and banged our heads against the wall’. (Academic Account Administrator)
	Inherited administrative norms	‘We can cure cancer and split the atom, but we can't hand people cash’? (Academic Account Administrator)

### 4.1 Benefits of Compensation

The strongest, most prominent theme across our research emphasizes the multiple benefits of appropriately compensating research participants. These processes build trust and respond to the principle of social beneficence, among others.

#### 4.1.1 Respect and Equity

Many participants emphasized that compensation is not just transactional; it is also symbolic, and meaningfully so. The process represents a sense of recognition, dignity and equity. For instance, one participant stated, ‘[Compensation is] not just a transaction. It's a statement of respect, recognition, and equity’ (Lived Expert). Another person told the group, ‘If you're going to pay a consultant, then you should respect me enough to pay me in just the same way. I see it as no different’ (Lived Expert). These views reframe compensatory payment not as a special perk or a helpful convenience, but as a moral imperative of basic fairness, and a form of validation for the expertise individuals bring to the research process.

#### 4.1.2 Honours Lived Experience as Expertise

Another subtheme within this broader discussion of compensation benefits is that lived experience is a legitimate form of expertise. The Lived Expert Task Group emphasized that such expertise warrants recognition equal to formal education or professional credentials. As one member stated, ‘There ain't nobody at this table that doesn't have, at minimum, the equivalent of a master's [degree] in the School of Hard Knocks. We may not have the [formal] degree, but we have experience that's more valuable’ (Lived Expert). This reframing challenges traditional knowledge hierarchies and highlights that lived experience offers unique, essential insight, especially in research affecting marginalized communities. In projects involving people with lived experience of homelessness, appropriate compensation acknowledges that expertise. Participants also compared their contributions to those of paid consultants, rejecting the idea that lived experience is less valuable: ‘Compensate me for my lived expertise, the way you would compensate a contractor… just because it's my life doesn't mean I don't get to be compensated for it’ (Lived Expert).

### 4.2 Benefits of Direct Cash Transfers

Throughout our meetings and public presentations, Lived Expert Task Group members repeatedly noted that beyond appropriate levels of compensation, there was a necessity for being paid in ‘cold hard cash’ for their time and expertise. Cash, as opposed to gift cards or other forms of compensation, had both practical and symbolic meaning for group members.

#### 4.2.1 Practicality of Cash Honoraria

Even when compensation is offered, the form it takes can create barriers. Participants described difficulties using gift cards, cashing cheques and accessing banks: ‘A lot of our people, our friends, our associates, may not have a bank account, may not have ID, may not have transportation to get to a bank’ (Lived Expert). One Lived Expert Task Group member recounted a prior project where compensation came as cheques that required travelling to pick them up and then to a bank to cash them, noting the mismatch between effort and reward: ‘You want me to go all around town, with all of these barriers, just to get my $60’? (Lived Expert). Cash avoids many of these obstacles and allows flexibility to cover gas, food, transportation and emergencies, which gift cards often cannot. Reflecting on gift card compensation, another member explained, ‘I couldn't spend those gift cards on the things I actually needed… those Amazon gift cards went to waste’ (Lived Expert).

#### 4.2.2 Participant Autonomy and Empowerment

There was a strong, repeated preference communicated during team meetings and public presentations for direct cash payments over gift cards or cheques. Participants described wanting agency over how compensation is used. The criticism of gift cards and cheques highlights barriers created by assumptions around trust and control: ‘When you give us a gift card, that's you telling me what my basic needs are. And I don't need you to tell me what my basic needs are. I know what I need, and I don't need that told to me’ (Lived Expert). Lived expert group members noted the inequity and paternalization inherent in non‐cash payments because of the limited control over where and how these forms of compensation can be used: ‘Nobody tells you what to do with your paycheck, so why is that different for me’? (Lived Expert).

Compensation was also described as affecting participants’ sense of self‐worth and motivation. Respectful, cash‐based compensation fosters confidence, empowerment and inclusion: ‘[Cash compensation] opens the door…so that we can take up space and own the space and own our voice’ (Lived Expert).

### 4.3 Barriers to Implementing Cash Payments

#### 4.3.1 Institutional ‘Red Tape’

Lived experts and university administrators acknowledged the bureaucratic resistance to cash payments due to perceived risks and institutional policies. These structures often reflect a lack of trust and systemic infantilization of people with lived experience. Academic account administrators have found ways to adapt to using cash payments, but acknowledged that dealing with cash is often more burdensome, risky and administratively complex than alternatives like gift cards. One account administrator noted the administrative challenges associated with providing cash compensation, but also noted that research proposals and participant responses were crucial in moving through administrative difficulties in providing cash:

They [university administration] said we couldn't use cash, that it was too risky. Well, we used your [university‐based researchers’] proposal, and we used these folks’ [Lived Experts] words and experiences to make the case that we had to use cash, and we banged our heads against the wall, but we ultimately got it done.

Academic account administrators also emphasized inconsistency among systems for research compensation, varying the processes for and successes of implementing direct cash compensation. In this research project, the total cash budget for the project was stored securely and inconspicuously in a departmental account administrator's office; for each meeting, a university researcher met with the administrator and withdrew the cash needed for the meeting, appropriately accounting for the remaining funds. Researchers asked that cash recipients sign for each cash payment to make sure that the amount received corresponded to the amount withdrawn from the account. Account administrators endorsed the process, and over the course of the project, ultimately became champions of using cash in these kinds of projects:

Really, I'm responsible for it if [the cash] is in my office locked up, and then you as the faculty are responsible for it getting to the participants. I think the way that you and I set it up was great.

(Academic Account Administrator)

Institutional red tape was not only associated with university structures, but of larger political economic structures as well. For instance, many on our Lived Expert team reside in permanent supportive housing that requires them to remain below a set low‐income threshold to keep their housing. Additionally, the US tax code requires reporting payments of more than $600 annually from universities to non‐employees; in practicality, this means that if Lived Expert team members received more than $600 from the university in a given calendar year, they would need to become an employee and file taxes. For many of our Lived Experts, these bureaucratic structures would have been prohibitive for them. Subsequently, participants argue that compensation must be context‐aware, including the need to navigate housing voucher limits, avoid penalizing people on benefits or accommodate their unique needs. The system must be flexible, compassionate, and just. ‘I understand I can also only get paid this much…but I should still be compensated potentially in other ways’ (Lived Expert).

#### 4.3.2 Inherited Administrative Norms

University administrators described that barriers for direct cash compensation for research participants perpetuate problematic norms about what kinds of research is valued, one administrator asking, ‘We can cure cancer and split the atom, but we can't hand people cash for helping out with our research projects’? (Academic Account Administrator). This quote reflects previous frustrations, as well as a perceived misalignment among institutional values and resources.

Despite institutional constraints, advocates within the university can challenge and find ways to navigate institutional red tape, showing that with persistence, systems can be changed, while still maintaining their rigour and accountability. Interviewed account administrators consistently framed this added labour as worthwhile due to the positive impact on participants’ autonomy and dignity, shared purpose with the research team in creating a respectful, flexible and person‐centred research process, and a belief in adapting institutional systems to better meet the needs of the community of concern. Account administrators recognized that removing institutional barriers can better serve research participants—particularly vulnerable populations—and help overturn problematic and outdated research norms. As one Lived Expert noted, in agreement, ‘We don't want to continue to contribute to incarceration of people through the thought process… We want to bring about normalcy’.

## 5 Discussion

Our research demonstrates the benefits of compensation, specifically of cash, for lived experts of homelessness. Further, we recognize and describe the institutional barriers to accessing and distributing cash for research participation. These findings underscore many of the paradigmatic approaches of Tuhiwai Smith [4] in that these practices of compensation are intertwined with academic research practices that often extract from, objectify, commodify and ‘other’ many communities, particularly those who are Indigenous or otherwise face issues of social and political marginalization [2]. Again, decisions regarding who receives payment, how much they are paid and in what form are not politically or morally neutral; rather, they are shaped by historical patterns of control, as well as epistemic and material forms of violence, often perpetuated through research practices [4], including research and engagement with people with lived experience of homelessness [32].

This study contributes to scholarship advocating for ethical, equity‐centred research practices, particularly regarding compensation for participants with lived experience of homelessness. Like other studies highlighting community advisory boards of people with lived expertise (e.g., [6, 10]), our study notes social and institutional challenges associated with these boards, even when outcomes benefit the group, researchers and broader community [29]. The findings emphasize that decisions about compensation—who is paid, how much and in what form—are not neutral or apolitical. They are embedded in long‐standing systems of control, inequality, structural knowledge suppression and physical oppression [4]. These dynamics are especially pronounced in homelessness research, where participation often implicates participants’ survival, dignity and autonomy.

Participants repeatedly emphasized that research compensation must be respectful, flexible, participant‐driven, institutionally supported and rooted in equity [2]. These principles call for a shift in institutional norms and ethical review, moving from restrictive, often paternalistic reimbursement (e.g., gift cards) to models recognizing participants as experts and workers. As one participant noted that research can ‘continue to contribute to incarceration of people through the thought process’, there is an urgent need to challenge systems that perpetuate marginalization by overlooking or undermining participants’ agency and lived realities [26].

This study invites reflection on how information from individuals with lived experience of homelessness is received and processed within academic and policy settings. We should question whether such research is treated merely as an intellectual exercise or recognized as a corporeal, relational and affective engagement. Honouring participants’ knowledge requires more than representation; it demands material reciprocity and structural accountability. Supporting participants’ expertise through direct cash payments is one way to advance this process. It may be best to understand that ‘paying research participants as workers resolve some ethical concerns about payment’ [12, p. 62]. This approach reframes participation not as charity or voluntary contribution, but as labour deserving fair compensation and even ongoing employment. Institutional reluctance to offer direct cash payments, often justified by ethical or legal concerns, can reflect discomfort with power redistribution and a resistance to challenge bureaucratic inertia.

### 5.1 Implications for Research

Several practical strategies align with this ethical stance and our research findings. First, research teams should include participant cash compensation in grant proposals and contracts to ensure funding for direct payments. This practice places responsibility on institutions to support and follow through with reimbursement processes approved by the granting agency. Second, teams must understand relevant tax laws, as some jurisdictions require reporting payments over $600 USD, which could unintentionally harm participants by triggering tax obligations or exceeding income thresholds. Teams should also identify additional mechanisms beyond standard procedures to provide cash compensation. Finally, institutional policies must be reviewed and updated to enable cash payments that meet participants’ needs rather than institutional convenience, with recipients advising on their own requirements. In our experience, participants almost always need and want cash. As Canham et al. [50] argue, failing to appropriately compensate insecurely housed participants reinforces the systems of exclusion, marginalization, stigmatization, discrimination and precarity that research often claims to address. Our findings also echo Tuhiwai Smith's [4] critique of research paradigms that extract from and commodify marginalized communities. Treating lived experience as data without material acknowledgement (e.g., direct, flexible payment) replicates the exploitation participants have long resisted.

In sum, compensating people with lived experiences of homelessness for their participation in research is not merely a transactional act; it is a moral, political and methodological imperative. Institutions must move beyond restrictive reimbursement models and toward practices that are equitable, respectful and participant‐centred [2]. Doing so means reshaping how research is funded, reviewed and implemented. It means listening to those most affected, not only as subjects of research, but as co‐creators of knowledge and rightful recipients of its benefits.

## Author Contributions

**Jeff Rose:** conceptualization, methodology, project administration, resources, writing – review and editing, writing – original draft, supervision, funding acquisition. **Steffine Amodt:** conceptualization, methodology, investigation, writing – review and editing. **Yixiao Burke:** conceptualization, methodology, investigation, writing – review and editing. **Sarah L. Canham:** conceptualization, methodology, supervision, funding acquisition, project administration, resources, writing – original draft, writing—review and editing. **Natalie Clark:** conceptualization, investigation, methodology, writing – review and editing. **Shawn Clay:** conceptualization, investigation, methodology, writing – review and editing. **Herbert Elliott:** conceptualization, investigation, methodology, writing – review and editing. **Rob Ferris:** conceptualization, investigation, methodology, writing – review and editing. **Ruby Gary:** validation, formal analysis, project administration, writing – original draft, writing – review and editing. **Lawrence Horman:** conceptualization, methodology, investigation, writing – review and editing. **Danielle Littman:** conceptualization, methodology, supervision, resources, project administration, funding acquisition, writing – original draft, writing – review and editing. **Maygan Martinez:** conceptualization, methodology, investigation, writing – review and editing.

## Ethics Statement

This research was determined to be exempt by the University of Utah IRB #00178772.

## Conflicts of Interest

The authors declare no conflicts of interest.

## Data Availability

The data that support the findings of this study are available on request from the corresponding author. The data are not publicly available due to privacy or ethical restrictions.
